# Is dietary fat associated with the risk of age-related macular degeneration? Protocol for a systematic review and meta-analysis

**DOI:** 10.1097/MD.0000000000019081

**Published:** 2020-04-24

**Authors:** Yunsheng Xu, Bo Lu, Yana Zhou, Shuxia Ren, Guoming Pang, Aijun Deng

**Affiliations:** a Second Affiliated Hospital of Shandong University of Traditional Chinese Medicine, Jinan, Shandong Province; b Shaanxi Traditional Chinese Medicine Hospital, Xi’an, Shaanxi Province; c Hubei Provincial Hospital of Traditional Chinese Medicine, Wuhan, Hubei Province; d School of Computer Science and Technology, Tianjin Polytechnic University, Tianjin; e Kaifeng Hospital of Traditional Chinese Medicine, Kaifeng, Henan Province; f Department of Ophthalmology, Affiliated Hospital of Weifang Medical University, Weifang, Shandong Province, China.

**Keywords:** age-related macular degeneration, dietary fat, meta-analysis, protocol, systematic review

## Abstract

Previous studies evaluating the association of dietary fat and risk of age-related macular degeneration (AMD) yield discrepant results. The objective of this systematic review (SR) and meta-analysis is to establish whether an association exists between dietary fat and AMD. This protocol was developed in line with the quality requirements of the Preferred Reporting Items for Systematic Review and Meta-Analysis Protocols (PRISMA-P) statement. PubMed and EMBASE will be searched for randomized controlled trials (RCTs), non-randomized trials (NRTs), cross-sectional studies, cohort studies, and case-control studies that evaluate the total incidence of AMD. The data extraction content and quantitative analysis will be carried out systematically. Newcastle-Ottawa Scale (NOS), the Cochrane risk of bias tool, and quality assessment tools will be used for quality assessment. This SR will synthesize evidence to determine if there is an association between dietary fat and AMD. The evidence would provide rationale for future research and serve as a basis for the development of future guidelines. Results are expected to be publicly available in mid 2020.

PROSPERO registration number: CRD42019137086.

## 1 Introduction

Age-related macular degeneration (AMD) is a third cause of blindness after cataract and glaucoma.^[1]^ The increasing size of the world's total elderly population, thus the effect of AMD continues to grow.^[2]^ AMD involves degenerative changes such as drusen, the changes in the retinal pigment epithelium, and subretinal neovascular membranes.^[3]^ Previous studies has been hypothesized that atherosclerosis of the vessels that supply the retina contributes to AMD, analogous to the mechanism underlying coronary heart disease (CHD).^[4–6]^ According to this hypothesis, dietary fats may be related to AMD. The association between dietary fat and AMD were initially based upon animal researches.^[7]^ Subsequently, randomized controlled trials (RCT) and many observational studies investigating the association have been done.^[8–10]^ Saturated fat, cholesterol,^[7,11]^ and trans unsaturated fats were shown to be positively correlated with the risk of CHD;^[12]^ polyunsaturated fatty acids were shown to be inversely related to the risk of CHD.^[13,14]^ Long-chain n3 fatty acids were abundant in the retina.^[15]^ Thus, these fats may have some associations with AMD. Although a substantial number of clinical studies have examined the relationship between dietary fat intake and AMD.^[8,9,16–18]^ The inconsistencies were observed in the different studies, the influence of dietary fat on AMD also remains unclear. It is necessary to firmly establish the effect of dietary fat intake on AMD from these studies.^[19]^ A systematic assessment on this subject has never, to the best of our knowledge, been performed before according to preferred reporting items for systematic review and meta-analysis (PRISMA) standard. All types of clinical studies across associations between dietary fat and AMD will be included without language limitation. Our objective is to conduct a SR to establish whether an association exists between dietary fat and AMD risk and, where possible, to quantify the relationship using the meta-analysis of individual studies.

## 2 Methods

### 2.1 Objectives

The objectives of this study is to synthesize the evidence about fat intake related to AMD in randomized and non-randomized trials, and cohort, cross-sectional, case–control studies that aim to estimate the effects of dietary fat on AMD risk.

### 2.2 Protocol registration

A protocol including the search strategy and methods of data analysis have been prepared according to the Preferred Reporting Items for Systematic Reviews and Meta-analysis (PRISMA). This SR and meta-analysis protocol is reported in line with the Preferred Reporting Items for Systematic Reviews and Meta-analysis Protocols (PRISMA-P) checklist.^[20]^ PROSPERO registration number CRD42019137086 (https://www.crd.york.ac.uk/prospero/display_record.php?ID=CRD42019137086)

### 2.3 Eligibility criteria

Studies will be included in SR and meta-analysis if they meet all of the following criteria:

(1) Randomized and non-randomized trials, and cohort, cross-sectional, case–control studies that aim to estimate the effects of dietary fat on AMD risk;
(2) Participant age ≥18 years;
(3) Dietary fats defined according to Directive 2002/46/EC of the European parliament and of the Council;
(4) Modalities of supplement intake: liquid, pill, capsule, tablet, drops, ampoule, powdered;
(5) The primary outcome: The total incidence of AMD;
(6) Report sample size, the number of events, and follow-up time for each group, report the hazard ratio or, provide sufficient details for this to be calculated.

### 2.4 Exclusion criteria

(1) Studies with a dietary co-intervention will be excluded;
(2) Studies with a drug intervention will be excluded;
(3) Studies with intravenous or parenteral administration will be excluded;
(4) Studies with pregnant or lactating women will be excluded;
(5) Studies with unreported follow-up time will be excluded.

### 2.5 Study types

Randomized and non-randomized trials, and cohort, cross-sectional, case–control studies are eligible for the SR and meta-analysis.

### 2.6 Search strategy

We will conduct searches in PubMed (from 1966) and EMBASE (from 1980). We will search for articles of original study by using the following search terms: diets [tiab] OR diet [tiab] OR dietary [tiab] OR dietetic [tiab] OR intake [tiab] OR eating [tiab] OR nutrition [tiab] OR nutrient∗. Furthermore, the key words closely related to fat will include “polyunsaturated fat∗” OR “unsaturated fat∗” OR saturated fat∗ “” OR lipid∗ [tiab] OR “monounsaturated fat∗”OR linoleic OR linolenic OR eicosapentaenoic OR arachidonic OR EPA OR DPA OR docosahexaenoic OR DHA OR docosapentaenoic. The outcome measures terms were consistent with measures used in previous SR and meta-analyses, including AMD and the synonyms of AMD. A search strategy will be performed for unpublished data. Moreover, the reference lists will be checked to search for further relevant studies. About language or publication year, there will be no restrictions.

### 2.7 Study selection

Two reviewers independently evaluated eligibility based on titles and abstracts of all retrieved studies. Full texts of all potentially eligible studies passing the title and abstract screening will be retrieved and examined independently by 2 reviewers in accordance with the abovementioned eligibility/exclusion criteria. Disagreements will be resolved by adjudication by the third reviewer.

### 2.8 Data extraction

After determination of the studies to be included, the following study data will be extracted: publication year, first author's last name, country, study design, study population, number of arms, sample size, duration, follow-up, participants’ sex and age, dietary fats, dose, administration, baseline risks (BMI, smoking, glycaemia, hypercholesterolemia, blood pressure, co-medications), AMD incidence and hazard ratios, drop-outs and withdrawals and funding. These variables will be extracted for all studies by the reviewer 1 (Xu) and the reviewer 2 (Lu), and GMP and AJD will arbitrate any disagreement and ensure that no errors occur during the review.

### 2.9 Risk of bias assessment

Methodological quality will be assessed with the risk of bias assessment tool from Newcastle-Ottawa Scale (NOS) and the Cochrane risk of bias tool. Selection bias performance bias (blinding of participants and personnel), detection bias (blinding of outcome assessment), reporting bias (selective reporting), attrition bias (incomplete outcome data), and rand industry bias will be assessed.

### 2.10 Strategy for data synthesis

We will present all the clinical evidence from the selected studies and then repeat certain analyses, excluding studies that do not meet certain quality criteria: clinical studies with a size of at least 30 cases for better statistical power; studies that excludes hysterectomies from the control group; and studies that adjust for important confounders, such as BMI. We will try to obtain missing data from the authors of the included studies (by email). Where possible, meta-analyses will be conducted to evaluate AMD risk compared with the lowest categories of fat intake.

### 2.11 Analysis of subgroups or subsets

Subgroup analyses will be conducted for studies that adjusted for fat intake and for those that did not. Subgroup analyses will also be stratified by the study design types, geographic areas and some confounding factors.

### 2.12 Quality of the evidence

The approach of GRADE will be used to rating quality of evidence.^[21]^

### 2.13 Dealing with missing data

We will attempt to obtain relevant missing data from the authors (by email).

## 3 Statistical analysis

The pooled odds ratios (ORs) with 95% CIs will be computed from the adjusted ORs and relative risk (RR) to measure the association between dietary fats intake and the risk of AMD. In pairwise meta-analyses, heterogeneity between trial results will be tested with a Cochran's Q test; a value for I^2^ of >50% considered to represent substantial heterogeneity.^[22]^ Meta-regression and subgroup analyses will be performed to explore the possible source of heterogeneity, such as geographic region, study design and sample size. Subgroup analyses will be performed to assess the potential effect of the modification of variables, including geographic region, the study type, fats subtype and dose. Begg funnel plots and Egger linear regression test will be performed to assess the publication bias. We also plan to perform sensitivity analyses for long-term intervention trials (≥12 months), low risk of bias trials, studies in elderly people (≥65 years), and trials in men and women. A value of *P* < .05 was considered statistically significant. All analyses were conducted using STATA software (version 12.0; StatCorp, College Station, TX) and a value of *P* < .05 was considered as statistically significant.

### 3.1 Start date and anticipated completion date

Now, this protocol has been established (as of October 2019). The first-stage (advanced search) will be completed by the end of December. It is anticipated that this systematic review will take 6 months to complete (November 30, 2019–May 30, 2020).

### 3.2 Ethics and dissemination

Ethical approval is not required in this protocol. We will collect and analyze data based on published studies, and since there is no patient involved in this study, individual privacy will not be under concerns. The results of this SR and meta-analysis will be disseminated to peer-reviewed journals.

## 4 Discussion

This study will determine whether an association exists between dietary fats intake and the risk of AMD. It is useful to public health policymakers and health professionals alike. Furthermore, this analysis will show which dietary fats, if any, are the most efficacious in the prevention of the evaluated AMD outcomes or cause the greatest harm. By considering all of the clinical evidence for the growing research into fats, our proposed SR will provide readers with a concise summary of fats-related studies and their impact on AMD.

## Author contributions

AJD is the guarantor of the article. The manuscript was drafted by YSX, BL and YNZ. YNZ and SXR developed the search strategy. YSX and GMP will independently screen the potential studies and extract data. YSX and BL will assess the risk of bias and finish data synthesis. GMP and AJD will arbitrate any disagreement and ensure that no errors occur during the review. All review authors critically reviewed, revised, and approved the subsequent and final version of the protocol.

Aijun Deng orcid: 0000-0001-6741-162X.
